# Genetic Diversity, Population Structure and Marker-Trait Association for 100-Seed Weight in International Safflower Panel Using SilicoDArT Marker Information

**DOI:** 10.3390/plants9050652

**Published:** 2020-05-21

**Authors:** Fawad Ali, Muhammad Azhar Nadeem, Muzaffer Barut, Ephrem Habyarimana, Hassan Javed Chaudhary, Iftikhar Hussain Khalil, Ahmad Alsaleh, Rüştü Hatipoğlu, Tolga Karaköy, Cemal Kurt, Muhammad Aasim, Muhammad Sameeullah, Ndiko Ludidi, Seung Hwan Yang, Gyuhwa Chung, Faheem Shehzad Baloch

**Affiliations:** 1 Department of Plant Sciences, Quaid-I-Azam University, Islamabad 45320, Pakistan; fawadali365@gmail.com (F.A.); hassaan@qau.edu.pk (H.J.C.); 2 Department of Field Crops, Faculty of Agriculture and Natural Science, Bolu Abant Izzet Baysal University, 14030 Bolu, Turkey; mbarut@cu.edu.tr (M.B.); sameepbg@gmail.com (M.S.); 3 Faculty of Agricultural Sciences and Technologies, Sivas University of Science and Technology, 58140 Sivas, Turkey; azharjoiya22@gmail.com (M.A.N.); tkarakoy@sivas.edu.tr (T.K.); mshazim@gmail.com (M.A.); 4 Department of Field Crops, Faculty of Agriculture, Çukurova University, 1000 Adana, Turkey; rhatip@mail.cu.edu.tr (R.H.); ckurt@cu.edu.tr (C.K.); 5 CREA Research Center for Cereal and Industrial Crops, 40128 Bologna, Italy; ephrem.habyarimana@crea.gov.it; 6 Department of Plant Breeding and Genetics, The University of Agriculture, Peshawar 25130, Pakistan; drihkhalil@gmail.com; 7 Science and Technology Application and Research Center (BİLTEM), Yozgat Bozok University, 66900 Yozgat, Turkey; ahmad.alsaleh@bozok.edu.tr; 8 Department of Biotechnology and Center of Excellence in Food Security, University of the Western Cape, Robert Sobukwe Road, Bellville 7530, South Africa; nludidi@uwc.ac.za; 9 Department of Biotechnology, Chonnam National University, Chonnam 59626, Korea; ymichigan@chonnam.ac.kr

**Keywords:** *Carthamus tinctorius*, genotyping by sequencing, germplasm characterization, GWAS, oilseed crop

## Abstract

Safflower is an important oilseed crop mainly grown in the arid and semi-arid regions of the world. The aim of this study was to explore phenotypic and genetic diversity, population structure, and marker-trait association for 100-seed weight in 94 safflower accessions originating from 26 countries using silicoDArT markers. Analysis of variance revealed statistically significant genotypic effects (*p* < 0.01), while Turkey samples resulted in higher 100-seed weight compared to Pakistan samples. A Constellation plot divided the studied germplasm into two populations on the basis of their 100-seed weight. Various mean genetic diversity parameters including observed number of alleles (1.99), effective number of alleles (1.54), Shannon’s information index (0.48), expected heterozygosity (0.32), and unbiased expected heterozygosity (0.32) for the entire population exhibited sufficient genetic diversity using 12232 silicoDArT markers. Analysis of molecular variance (AMOVA) revealed that most of the variations (91%) in world safflower panel are due to differences within country groups. A model-based structure grouped the 94 safflower accessions into populations A, B, C and an admixture population upon membership coefficient. Neighbor joining analysis grouped the safflower accessions into two populations (A and B). Principal coordinate analysis (PCoA) also clustered the safflower accessions on the basis of geographical origin. Three accessions; Egypt-5, Egypt-2, and India-2 revealed the highest genetic distance and hence might be recommended as candidate parental lines for safflower breeding programs. The mixed linear model i.e., the Q + K model, demonstrated that two DArTseq markers (DArT-45483051 and DArT-15672391) had significant association (*p* < 0.01) for 100-seed weight. We envisage that identified DArTseq markers associated with 100-seed weight will be helpful to develop high-yielding cultivars of safflower through marker-assisted breeding in the near future.

## 1. Introduction

Safflower (*Carthamus tinctorius* L.) belongs to the family *Compositae* and it is a self-pollinated crop with a genome size of about 1.4 GB [1]. Safflower is cultivated in 20 different countries of the world on a total cultivated area of 1,140,002 hectares that produces approximately 948,516 tons [2]. It is grown as an important industrial crop for different purposes, which include extraction of edible oil, production of dyes, and several uses in the pharmaceutical industry [3,4,5]. Safflower has better adaptation to stress conditions such as salinity and drought, although it produces oil in lower quantity than other oilseed crops [6]. Safflower also gained importance because it has the capability of biofuel production [7]. *Carthamus* species have been utilized since the pre-historic period as its archeological remains were found at sites in Syria since 7500 BC. Safflower was distributed from its center of domestication (i.e., Syria) to linked regions comprising Egypt, the Aegean region and southern Europe [8]. 

Safflower is considered an underutilized crop in comparison to other oilseed crops such as soybean, rapeseed and sunflower [3]. Key factors contributing to its underutilized status include lower oil content and seed yield, insect pest susceptibility, and lower resistance to diseases, which decrease safflower productivity and quality [9]. Narrow genetic diversity of local and traditional varieties necessitate exploration of the genetic diversity of the available germplasm by collecting accessions from different geographies worldwide. Such collections will provide information that will aid safflower conservation and utilization in the future [10]. Domestication of safflower has bottlenecked its genetic diversity, which greatly reduced its adaptive potential against different environmental stresses [10,11]. Exploration of genetic diversity is regarded as an important tool yielding a good source of trait variations [12,13]. Genetic diversity present in the germplasm contributes to execution of successful breeding programs [14]. Germplasm possessing diverse traits may be helpful in breeding programs for the development of elite cultivars [15,16]. Characterization of crop germplasm also helps in food security through the identification of novel genetic variations [11,12,13,14]. The Food and Agriculture Organization described the decrease of crop genetic diversity as one of the important factors that negatively impact food security and the environment [17]. The availability of limited information regarding genetic diversity and lack of the efficient genomic tools are considered as hampering factors to the improvement of safflower agronomic traits in breeding programs. Great emphasis on the molecular characterization and development of efficient molecular markers in safflower is required to accelerate safflower breeding activities [18,19,20]. Safflower genetic diversity using different molecular markers has been estimated [2,10,18,21,22,23,24,25,26,27]. 

Next generation sequencing technologies, such as genotyping by sequencing (GBS) and multiplex sequencing, aid in the generation of massive genetic data for various applications [28]. Application of the current polymerase chain reaction (PCR)-based marker technologies—aiming at whole genome analysis for association studies, construction of genetic maps, assessment of the collected germplasm for large scale molecular evaluation, and genome wide selection of the desirable alleles—are not attainable due to consumable and labor costs [28]. The application of DNA hybridization-based technologies like some SNP technologies and Diversity Arrays Technology are more suitable for such purposes. Hassani et al. [29] implemented DArTseq technology to assess genetic diversity in 89 safflower accessions originating from different countries of the world. They applied 1136 silicoDArT markers along with 2295 SNPs in their investigation. 

Linkage analysis, also known as QTL mapping, helps in the identification of genomic regions controlling complex plant traits. QTL mapping is a time-consuming technique that needs mapping populations to be developed from bi-parents. QTL mapping captures less allelic variation utilizing bi-parental populations due to the very low rate of occurrence of recombination events and low mapping resolution [30]. Association mapping is a more efficient and faster technique, which provides higher resolution of complex plant traits in comparison to QTL mapping. Association mapping emerged as a promising technique to avoid limitations present in QTL mapping [31,32]. Relationships between plant traits and genetic polymorphisms observed in a heterogeneous assembly of distinct individuals, utilizing naturally occurring recombination events, aid in fine scale mapping of traits. Ambreen et al. [21] and Ebrahimi et al. [33] identified marker-trait associations in safflower, utilizing SSR and AFLP marker systems, respectively. Yield in any crop is the most important trait that is polygenic and difficult to measure and improve as it is highly influenced by other contributing traits. Thus, indirect selection for yield is highly preferred in comparison to direct selection focusing on the yield-contributing traits [34]. This study aimed at the establishment of the usefulness of silicoDArT markers to investigate phenotypic and genetic diversity, population structure and marker-trait associations for 100-seed weight. To attain these aims, we implemented a total of 12232 silicoDArT markers detected by a DArTseq approach of genotyping by sequencing in a safflower panel collected from 26 countries.

## 2. Results

### 2.1. Phenotypic Data Evaluation

**Figure 1:**
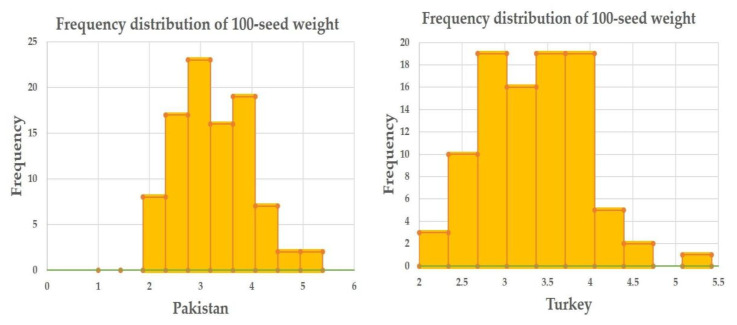
Frequency distribution chart for 100-seed weight for both studied locations (Pakistan and Turkey).

During this study, 100-seed weight at both locations (Pakistan and Turkey) was recorded with the help of an electronic seed counter and weighing balance by taking undamaged and fully matured seeds. Analysis of variance (ANOVA) for both locations revealed highly significant differences among the studied safflower accessions for 100-seed weight (Table 1). Minimum, maximum, and mean values for 100-seed weight reflected sufficient phenotypic variation in the studied safflower panel (Table 2). Overall 100-seed weight ranged from 2.17 to 5.32 g with an average of 3.33 g. This reflects the presence of genetic variability and suggests that the safflower accessions studied here are suitable for association analysis. Mean 100-seed weight in Pakistan was comparatively lower than 100-seed weight obtained from Turkey (Table 2). Safflower accessions Egypt-5, China-3 and China-5 recorded superior 100-seed weight at both locations (Supplementary Table S1). Frequency distribution revealed a normal distribution of 100-seed weight at both locations (Pakistan and Turkey) and better mean performance of the safflower accessions for 100-seed weight in Turkey compared to Pakistan (Figure 1).

**Figure 2:**
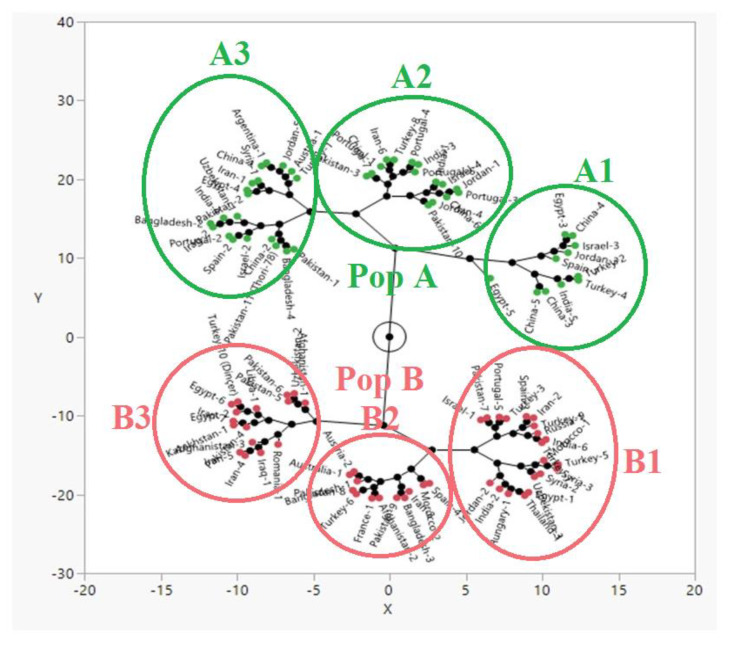
Constellation plot for 100-seed weight in international safflower panel.

The implemented constellation plot clearly divided international safflower panel into two populations on the basis of their 100-seed weight. A total of 46 and 48 accessions clustered in population A and population B, respectively (Figure 2).

### 2.2. DArTseq Profiling by GBS

**Figure 3:**
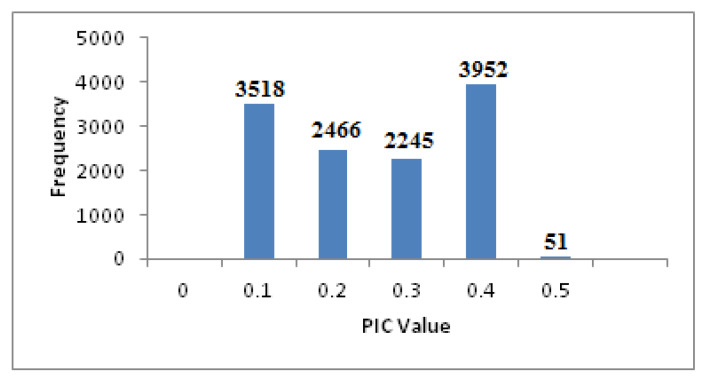
Frequency distribution of PIC values of 12232 silicoDArT markers.

DArTseq profiling of 94 safflower accessions resulted in a total of 29,048 silicoDArT markers. This dataset was filtered by accounting markers having less than 5% missing data, polymorphism information content (PIC) value of 0.10 to 0.50, call rate greater than 0.81, and 100% reproducibility, to retain 12,232 high quality markers for further analysis. Figure 3 shows the distribution of PIC values of the filtered silicoDArT marker dataset. The whole safflower panel revealed maximum and minimum PIC values of 0.50 and 0.10 respectively, with an average of 0.31. Highest and lowest call rate values of 1.00% and 0.81%, with an average of 0.93%, were obtained through the whole safflower panel.

### 2.3. Genetic Diversity and Population Structure Analysis in Safflower Panel

Various diversity parameters such as observed number of alleles (1.99), effective number of alleles (1.54), Shannon’s information index (0.48), expected heterozygosity (0.32), and unbiased expected heterozygosity (0.32) reflected a good level of genetic variation in the studied germplasm (Table 3, Supplementary Table S2). Maximum genetic distance (0.76) was found between Egypt-2 and India-2 accessions, while mean genetic distance for the entire safflower population was 0.50 (Supplementary Table S3). Diversity indices were investigated on country basis, and Pakistan and Turkey revealed the existence of maximum percentage of polymorphic loci and high diversity parameters from the rest of countries (Table 3).

**Figure 4:**
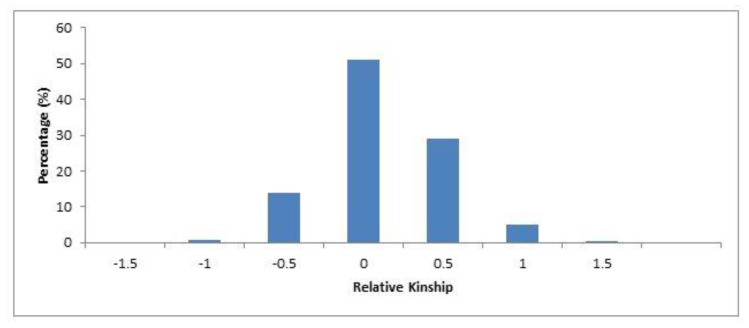
The proportion of pairwise kinship coefficients in international safflower panel.

**Figure 5:**
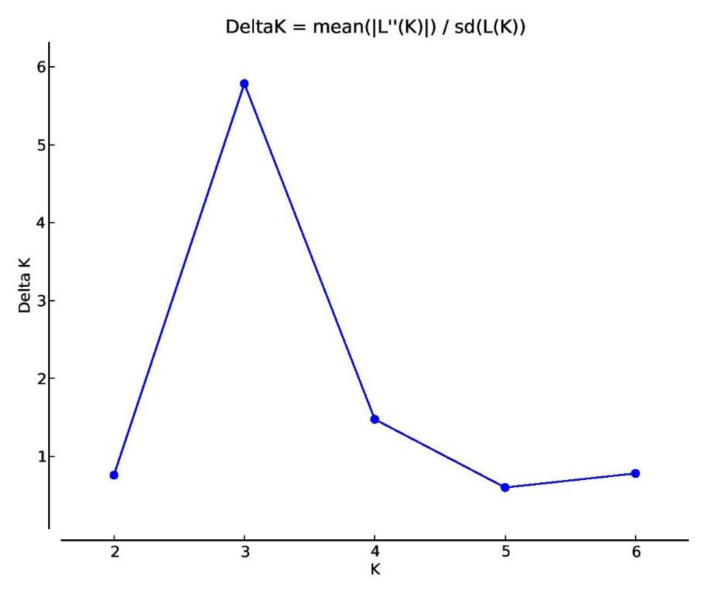
Delta K for the entire safflower population indicating the presence of three subpopulations at K = 3.

Pairwise kinship coefficients ranged from −1.45 to 1.24 for the entire safflower panel. A total of 51.17% kinship values ranged from −0.40 to 0.4. 99% of the kinship coefficient values, which ranged from 0.60 to 1.00; however, 0.21% of the kinship coefficients ranged from 1.10 to 1.30, respectively (Figure 4). Analysis of molecular variance (AMOVA) revealed the division of the total variation into two stratum; i.e., among countries (9%) and within country group (91%) (Table 4). The ΔK peak at K = 3 in the structure analysis revealed that the genetic structure of the 94 safflower accessions is divided into three groups (Figure 5). 

**Figure 6:**
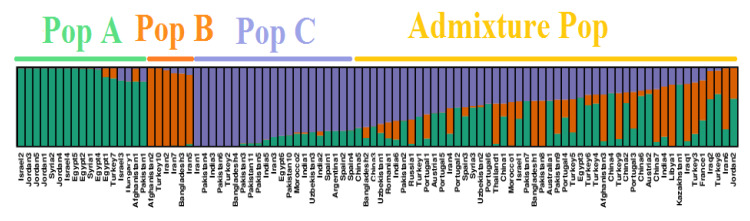
Structure-based clustering of the 94 safflower accessions using silicoDArT molecular markers.

**Figure 7:**
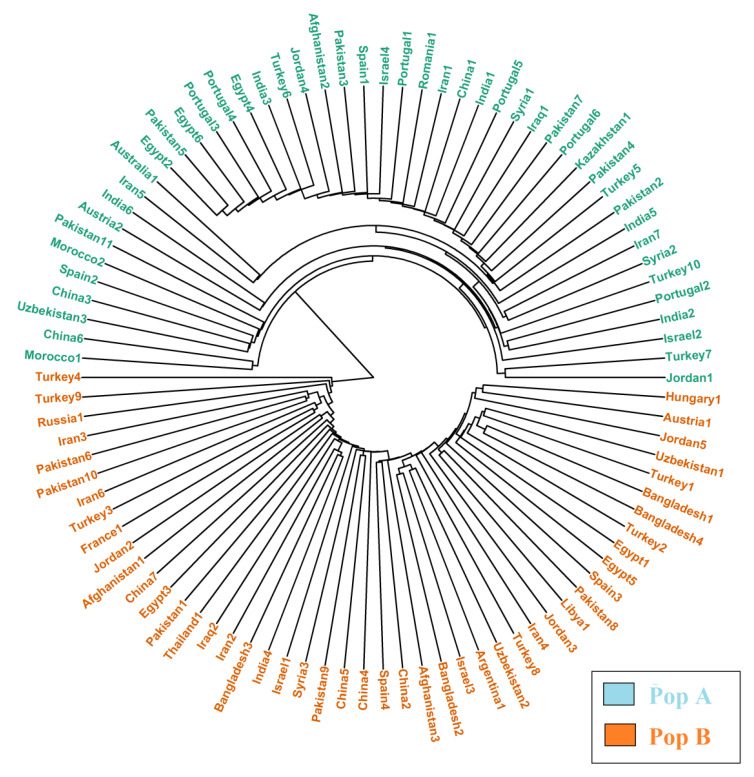
Neighbor joining-based clustering of the 94 safflower accessions using silicoDArT molecular markers.

**Figure 8:**
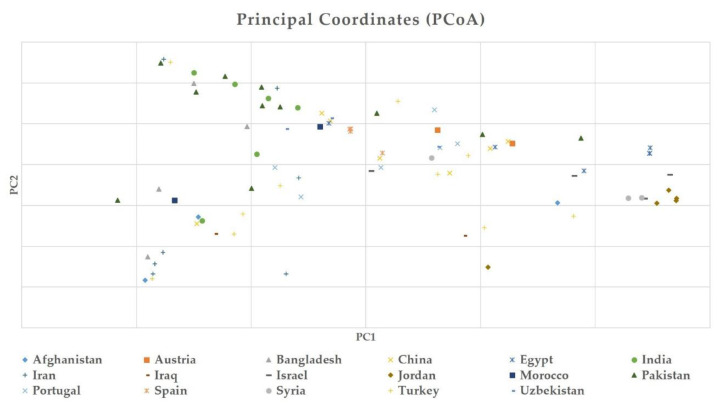
Principal coordinate analysis (PCoA) of the 94 safflower accessions using silicoDArT molecular markers.

The Bayesian clustering model grouped the international safflower panel into three main populations implemented in STRUCTURE software on the basis of membership coefficient: 17 accessions in population A, 6 accessions in population B, and 21 accessions in population C. The remaining 50 accessions were clustered as admixture population (Figure 6). Clustering of the safflower accessions within the same population revealed membership coefficients of either 80% or greater than 80%. The Neighbor Joining analysis divided the 94 safflower accessions into two populations (A and B), each containing 47 accessions (Figure 7). PCoA was also performed and results showed that collection countries played some role in the clustering and an admixture of accessions was found as well, which had a similar structure clustering (Figure 8).

### 2.4. Marker-Trait Associations and Putative Candiadate Genes Identification for 100-Seed Weight

**Figure 9:**
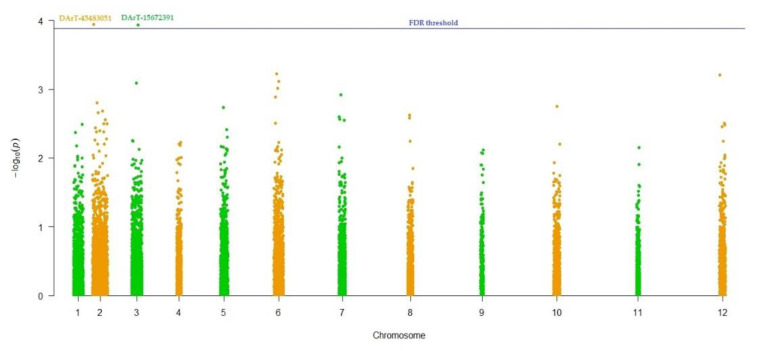
Pseudo manhattan plot for 100-seed weight in world safflower panel. DArT-45483051 and DArT-15672391 were considered statistically (FDR thresholds *p* = 0.01) associated with this trait.

The MLM (Q + K) model was performed to assess marker-trait associations for 100-seed weight in the international safflower panel. DArT-45483051 and DArT-15672391 showed marker-trait association for 100-seed weight by revealing statistically significant association (*p*-value; 1.17E-04 and 1.15E-04), respectively (Figure 9). DArT-45483051 and DArT-15672391 markers were present on supposedly chromosome 2 and 3, respectively. DArT-45483051 and DArT-15672391 markers contributed a total of 17.40% and 18.60% variation for 100-seed weight, respectively (Table 5). BLAST search against NCBI for DArT-45483051 resulted in AT1G01040 as a putative candidate gene. A search using the DArT-15672391 marker resulted in the retrieval of AT5G58040 as a putative gene for this marker. 

## 3. Discussion

The studied safflower panel revealed statistically significant differences for 100-seed weight. Analysis of variance (ANOVA) confirmed the statistically significant genotypic effects in both locations (Table 1). These results were found to be in line with El-Lattief et al. [35] as they also found statistically significant genotypic effects for various agronomic traits of safflower, including 1000-seed weight. Frequency distribution for 100-seed weight at both locations (Pakistan and Turkey) was also calculated for a better understanding of the distribution of data. There was a normal frequency distribution of 100-seed weight at both locations. Frequency distribution revealed that only one safflower accession (Egypt-5) resulted in average 100-seed weight of > 5.00 g at both locations (Figure 1). Overall mean (3.29 g) and range of 100-seed weight in this study were higher than that reported in a previous study [36]. Mean and range of 100-seed weight obtained from Turkey were slightly higher than those obtained from Pakistan. Köse et al. [37] ascribe variations in seed weight to be a result of the reaction of genotypes to different environments. Our ANOVA also confirmed that there is a statistically significant genotypic effect on 100-seed weight. Constellation plot clustered the whole germplasm into two main populations, i.e., 46 and 48 accessions present in population A and B based on their 100-seed weight (Figure 2). Further division of accessions into subpopulations occurred. i.e., A1, A2, A3, B1, B2, and B3. Subpopulation A1 clustered only those 12 safflower accessions having 100-seed weight >4 g. Subpopulation A2 clustered safflower accessions having 3.70 to 4.00 g 100-seed weight. Accessions having 100-seed weight range 3.24 to 3.59 g clustered together, making subpopulation A3. Subpopulation B1 clustered safflower accessions having 100-seed weight ranging from 3.13 to 3.29 g. Accessions having 100-seed weight in a range of 2.64 to 2.85 g clustered together to make subpopulation B2. All accessions having 100-seed weight <2.60 g were present in subpopulation B3. As it is obvious from the above discussed results, 12 accessions present in the A1 subpopulation have higher 100-seed weight than the rest of the accessions. Therefore, these accessions can be used for breeding activities of safflower for high yield.

DArTseq technology gained the attention of scientists globally due to low cost and high throughput nature. DArTseq technology has been used to explore the genetic diversity and population structure of different crops with a large number of entries and complex genomes [38,39]. Hassani et al. [29] used DArTseq technology to explore genetic variations in a world panel of 89 safflower genotypes of diverse origin. The safflower panel utilized in their investigation is different from our panel except one accession, i.e., Afghanistan-1. During this study we also aimed to explore the phenotypic and genetic diversity, population structure and marker-trait association in an international safflower panel using silicoDArT markers. Hassani et al. [29] used 1136 silicoDArT and 2295 SNP markers, while we used a higher number of markers (12232) for the molecular characterization. Moreover, Hassani et al. [29] used germplasm from 12 countries, while we included germplasm from 26 countries to explore population structure more extensively.

Polymorphism information content (PIC) value is a measure of polymorphism which provides information regarding the genetic diversity or DNA segment in a studied population, and indicates the allele’s evolutionary pressure and mutations that occurred at a locus over a time period. The range of the PIC value (0.10 to 0.50) obtained in this study suggests the existence of a high level of genetic variation that might be derived utilizing a large number of good quality markers in a diversified safflower panel. An average PIC value of 0.31 across all the silicoDArT markers was obtained during this study. PIC values were distributed asymmetrically and were skewed towards the lower values. More than 50% of the implemented silicoDArT markers revealed a PIC value of more than 0.30, which indicates the informativeness and usefulness of these markers for genetic diversity, population structure, and marker-trait association in safflower.

Diversity parameters including observed number of alleles (Na), effective number of alleles (Ne), Shannon’s information index (I), expected heterozygosity (He), and unbiased expected heterozygosity (uHe) for the entire population of 94 safflower accessions, which were 1.99, 1.54, 0.48, 0.32, and 0.32, respectively. Previous use of different gel-based marker systems obtained lower diversity metrics values than our current results from the silicoDArT marker system [2,18,40,41]. The most prominent reason for getting good diversity results is likely due to higher capability of the silicoDArT marker system in comparison with other gel-based marker systems. Differences in the experimental materials might also be another reason of revealing higher polymorphism in this study. Furthermore, results of diversity indices on the basis of collection countries revealed the highest polymorphism and genetic diversity for safflower genotypes from Pakistan and Turkey, while the lowest polymorphism and genetic diversity was obtained for safflower accessions originating from Iraq and Morocco. In a similar way, the highest mean genetic distance was observed for accessions originating from Turkey, and followed by India, Austria and Uzbekistan. The lowest mean genetic distance was observed for accessions originating from Jordan, followed by Spain (Table 3).

The Jaccard coefficients of genetic distance resulted in a mean value of 0.50 for the entire population of 94 safflower accessions. A maximum genetic distance was proposed between safflower accessions Egypt-2 and India-2, followed by Egypt-5 and India-2 with genetic distance values of 0.76 and 0.76, respectively. The highest genetic similarity was recorded between safflower accessions Spain-1 and Spain-2, with a genetic distance value of 0.14. The presence of higher genetic similarity between safflower accessions is possibly because of their origin from common parents (Supplementary Table S3). Safflower accessions containing desirable plant traits can be integrated in different breeding programs to aid in the development of superior cultivars [2]. The most diverse safflower accessions identified (Egypt-2, India-2, and Egypt-5) during the current evaluation can be recommended as candidate parental lines for future safflower breeding activities. The inferences obtained from kinship coefficient estimations with silicoDArT markers are robust to population structure. Negative kinship coefficients were also observed, suggesting an unrelated relationship between the safflower accessions. The close relatives can be inferred fairly reliably based on the estimated kinship coefficients. Thus, it is suggested that most of the safflower accessions were less related, having kinship coefficients of either 0 or below 0 (Figure 4). Analysis of molecular variance (AMOVA) revealed the division of the total variation into two strata, i.e., among countries and within country. A total of 91% of the genetic diversity was present within country group (Table 4). This is supported by Hassani et al. [29], where the majority of genetic variation among accessions within populations was obtained. The presence of a higher level of genetic variation within populations can be attributed to gene flow, which depends on the informal seed exchanges between farmers of different ecological zones [42].

### 3.1. Genetic Structure and Diversity in Safflower Panel

The three clustering algorithms important to genetic diversity and population structure analysis (model-based structure, Neighbor Joining, and PCoA) were implemented and revealed that the safflower accessions were successfully grouped by the silicoDArT markers based on geographical regions. Among the three clustering algorithms, more preference was given to the model-based structure algorithms. The reason for giving such a high preference to the structure is that this algorithm revealed more robustness in the previous works [43,44]. Structure algorithm divided the whole germplasm panel into four genetic populations: population A, B, C, and an admixture population. These different populations will aid in the selection of the parental accessions, which can used to design and conduct various crossing combinations for safflower genetic improvement (Figure 6).

Grouping of the safflower accessions obtained from structure analysis was based on the geographical origins of germplasm. Population A clustered safflower accessions originating from Israel, Jordan, Syria, Egypt, Turkey, Hungary, Afghanistan, and Pakistan. Out of 17 of these accessions, 13 belong to the geographical locations situated in the Mediterranean region. Safflower accessions from the Mediterranean region clustered together and revealed their genetic similarity, and possibly shared a similar parentage. Clustering of the safflower accessions from Mediterranean countries proposes this region as a center of safflower domestication, with Syria having a predominant role [8]. Population B clustered safflower accessions from the South Asian countries, i.e., Afghanistan, Iran and Bangladesh, but not Turkey. Population C revealed safflower accessions from Iran, Pakistan, India, Turkey, Bangladesh, Egypt, Morocco, Uzbekistan, Spain, and Argentina. All four populations clustered accessions from Turkey. Our results are strongly supported by Hassani et al. [29] as they observed that safflower accessions from the Asian continent like Pakistan, Iran, Turkey, and India were found genetically similar and grouped into the same cluster. Clustering of the safflower accessions originating from the Mediterranean region to other localities suggested the distribution of safflower accessions from the Mediterranean region to other geographies. Turkey signifies a high level of biodiversity and differentiation center for safflower among the continents, thus reflecting a key role in the connection of different continents (Asia and Europe) with each other [15], and might possibly have played a role in the distribution of safflower from its domestication center. Our current findings are also supported by previously conducted archeological and molecular characterization studies using safflower accessions with its wild progenitors. These studies concluded the domestication of safflower in Fertile Crescent and its distribution to other parts of the world, i.e., Europe, Africa, the Middle and Far East [45,46]. All safflower accessions with a membership coefficient below 80% were classified as admixture populations. Accessions from countries such as Afghanistan, Australia, Austria, Bangladesh, China, Egypt, France, India, Iran, Israel, Iraq, Jordan, Kazakhstan, Libya, Morocco, Pakistan, Portugal, Romania, Russia, Syria, Thailand, Turkey, and Uzbekistan were clustered in the admixture population. The representation of safflower accessions from such a wide range of countries in the admixture population might be due to their use in international breeding programs. Other important factors like mutation, migration and selection by humans might also be responsible for the occurrence of admixture populations in safflower accessions of different origin [21,27].

Neighbor joining analysis divided the studied germplasm into two populations based on their geographical origin (Figure 7). Structure-based clustering of the 94 safflower accessions was also greatly supported by the principal coordinate analysis (PCoA) with silicoDArT markers information. PCoA resulted in clustering of safflower accessions on the basis of their geographical origins (Figure 8). Clustering results by PCoA were also supported by the structure, as admixture of accessions was also observed in the PCoA clustering as well.

### 3.2. Marker-Trait Associations for 100-Seed Weight

Seed yield is a complex trait with many underlying factors contributing to it. Comprehensive understanding of the relationship between seed yield and other contributing traits is crucial to the process of selection and ultimately to crop improvement [47]. In safflower, 100-seed weight is regarded as an important yield trait. Seed weight is proposed as an important trait to the process of selection in safflower breeding programs [48,49,50]. Furthermore, Chaudhary [48] observed the positive effects of 1000-seed weight on seed yield in safflower. Identification of loci influencing important plant morpho-agronomic traits is a prerequisite to marker-assisted breeding for enhancement of crop productivity. Our current investigation involved the identification of two silicoDArT markers (DArT-45483051 and DArT-15672391) associated with 100-seed weight (Table 5). Earlier studies reported different loci/markers linked with 100-seed weight. Ambreen et al. [21] reported two loci (NGSaf_306 and NGSaf_309) associated with 100-seed weight utilizing SSR markers. Mirzahashemi et al. [51] identified one QTL (qThsw5) associated with 100-seed weight.

The BLAST search against DArT-45483051 marker resulted in AT1G01040, a putative candidate gene. AT1G01040 is highly associated with seed embryo health and embryo shape at seed maturity and ovule development [52]. Therefore, the marker DArT-45483051 associated with 100-seed weight can be suggested for marker-assisted breeding of safflower for this trait. The BLAST search against DArT-15672391 retrieved a gene (AT5G58040) that encodes the pre-mRNA polyadenylation factor FIP1. Recently, Téllez-Robledo et al. [53] explored the role of polyadenylation factor FIP1 for plant development and root response to abiotic stresses. Polyadenylation factor FIP1 plays an important role in the plant embryo cotyledonary stage of development. Paez-Garcia et al. [54] established that better root growth ultimately contributes to higher crop yield. Based on the role of polyadenylation factor FIP1 during the plant embryo cotyledonary stage, AT5G58040 should be considered as having a potentially important role in seed weight. The studied germplasm reflected a wide range of phenotypic variations for 100-seed weight. Moreover, various genetic diversity indices also confirmed the existence of higher polymorphism in the evaluated germplasm. Characterization of germplasm provides us with an opportunity to unlock the novel genetic variations that can be utilized for breeding purposes [55,56,57]. This is a pioneer study concerning the investigation of marker-trait association for 100-seed weight for safflower using GBS analysis. We believe that these identified markers can be utilized in safflower marker-assisted breeding in order to develop cultivars with improved yield.

## 4. Materials and Methods

### 4.1. Plant Materials and Phenotypic Evaluation 

A total of 94 safflower accessions originating from 26 countries were used as plant materials in this study. Seeds of the evaluated germplasm were provided by the United States Department of Agriculture (USDA) (Supplementary Table S4). The experimental materials were sown at two diverse locations, i.e., Pakistan and Turkey. The dirst experiment was conducted at the National Agricultural Research Center (Pakistan), whereas the second experiment was conducted at the research and experimental area of Bolu Abant Izzet Baysal University (Turkey) during 2016–2017 and 2018, respectively. Field experiments were performed by implementing an augmented block design. Seeds of each safflower accession were planted in elementary plots with a row length, inter-row and intra-row spacing of 3 m, 50 cm, and 10 cm respectively. A total of 10 plants for each accession were maintained for the phenotypic characterization. Thori-78 and Dinçer were included as check cultivars. Di-ammonium phosphate (DAP) and ammonium sulfate were applied as a source of fertilizer, while standard cultural practices were performed at both locations. Safflower accessions were harvested at their proper maturity at both locations. Measurement of 100-seed weight was done with the help of an electronic seed counter by taking undamaged and fully matured seeds randomly in triplicate.

### 4.2. Genomic DNA Isolation

To extract the genomic DNA from each accession, fresh, healthy and young leaves were harvested and kept frozen in the laboratory at −80 °C. DNA isolation of each safflower accession was performed utilizing the bulk of leaves from 10 individuals. The individuals used for the purpose of DNA isolation were from plants from the original seeds from the gene bank. DNA isolation was performed according to CTAB protocol [58] and a specific protocol suggested by Diversity Arrays Technology [59]. DNA concentration was estimated with agarose gel (0.80%) and was then confirmed with NanoDrop (DeNovix DS-11 FX, USA). For the genotyping by sequencing (GBS) analysis, DNA was diluted and a 50 ng.μl^−1^ DNA concentration was maintained. The prepared DNA samples were sent to Diversity Array Technology Pty, Ltd., Bruce, Australia, for DArTseq analyses of GBS [60].

### 4.3. DArTseq-Generated SilicoDArT Marker Analysis

DArTseq technology is a complexity reduction method and next generation sequencing platform [61]. DArTseq facilitated the selection of the genome fractions containing active genes associated with agronomically important plant traits [62]. Digestion/ligation reactions were used for the processing of DNA samples following the method described by Kilian et al. [63]. Mixed fragments (PstI–MseI) were amplified by performing 30 rounds of PCR cycles. Details of silicoDArT markers analysis can be found in earlier studies [63,64]. 

### 4.4. Statistical Analysis

#### 4.4.1. Phenotypic Data Analysis

Online software developed by Rathore et al. [65] for statistical inferences of augmented block design was used. Analysis of variance (ANOVA) for both locations was calculated through SAS 9.3 version [66]. Data recorded on 100-seed weight of both field experiments was averaged and used to calculate parameters like minimum, maximum, mean, standard deviation, and frequency distribution utilizing statistical software XLSTAT (Addinsoft, 2018) [67]. The cluster constellation plot for 94 safflower accessions was constructed through JMP 14.1.0 statistical software (2018, SAS Institute Inc., Cary, NC, USA).

#### 4.4.2. DArTseq Markers Analysis

All images were analyzed from the DArTseq platform using DArTsoft v.7.4.7 (DArT P/L, Canberra, Australia). SilicoDArT are dominant markers that were detected through DArTseq and scored using the binary fashion, where 1 or 0 represent presence or absence of the restriction fragment in the genomic representation of each sample, respectively [12,68]. Screening of the markers was done with various parameters including call rate, polymorphism information content (PIC) and reproducibility being considered. Markers with PIC, reproducibility and call rate lower than 0.10, 100% and 0.80% were ignored during bioinformatics analyses to avoid false inferences.

#### 4.4.3. Genetic Diversity Analyses

The proportion of shared alleles that were obtained from silicoDArT markers were used to compute the genetic distances among the safflower accessions using Jaccard's coefficients of genetic distance. Important diversity metrics such as observed number of alleles (Na), effective number of alleles (Ne), Shannon’s Information Index (I), expected heterozygosity (He), and unbiased expected heterozygosity (uHe) were estimated for the entire population using GenAlEx 6.5 [69]. The kinship coefficients between safflower accessions were calculated with hierfstat R package to investigate the pairwise relationships of the 94 safflower accessions. Analysis of molecular variance (AMOVA) was computed with GenAlEx 6.5 [69] considering total variation into two strata, i.e., among countries and within countries.

Population structure of the studied safflower accessions was evaluated with STRUCTURE software (version 2.3.4; [70]). The most suitable number of clusters (K subpopulations) ranging from 1 to 10 was determined applying STRUCTURE software following the protocol of Evanno et al. [71]. For each K value and for each run, 10 independent runs were set. The initial burn-in period was set to 500 with 500,000 MCMC (Markov Chain Monte Carlo) iterations with no prior information on the origin of individuals. The most probable number of subpopulations was investigated by following the methodology suggested by Evanno et al. [71]. Each accession was assigned to a specific population on the basis of a membership coefficient. The PCoA was performed with GenAlEx 6.5 [69], while the Neighbor Joining tree was constructed with hierfstat R package in R statistical software. The populations obtained from the Neighbor Joining and PCoA were named and colored with the same clusters pattern identified with model-based Structure algorithm for coherence purposes.

#### 4.4.4. Functional Analysis for Putative Candidate Gene Identification

To investigate the putative candidate genes, sequences of identified silicoDArT markers were used to perform BLAST searches against the National Center for Biotechnology Information (NCBI) [72], and the Phytozome V.12.1 [73] database. Moreover, detailed information about putative candidate genes was obtained from the TAIR database [74].

#### 4.4.5. Genome-Wide Association Mapping for 100-Seed Weight

A Mixed linear model (MLM, Q + K) approach was applied to inspect marker-trait associations (MTAs) via TASSEL 5.0.5 [75]. The population and family structure were corrected utilizing Q-metrics (Q) and kinship (K) during association analysis, as suggested by Nadeem et al. [76,77]. Scaled identity was utilized to detect kinship matrix by the descent method applied in TASSEL 5.0.5 [75]. In the results of association analysis, the *p* value signifies the relatedness of a marker with the associated trait, and R^2^ reflects the proportion of phenotypic variation resulting from a significant marker [78]. SilicoDArT markers with Bonferroni and FDR thresholds *p* = 0.01 were taken as significantly associated with the 100-seed weight. A Pseudo-Manhattan plot was developed using the qq-man R Package in the R 4.0.0 statistical software [79]. 

## 5. Conclusions

The current evaluation revealed a good level of phenotypic and genetic diversity in the studied safflower panel from the silicoDArT markers information. Analysis of variance (ANOVA) revealed the highly significant genotypic effect for 100-seed weight. Frequency distribution resulted in a normal distribution for 100-seed weight across the two locations (Pakistan and Turkey). The mean and range of 100-seed weight obtained from Turkey was higher than that from Pakistan. Analysis of molecular variance (AMOVA) revealed the division of total variations into two stratum i.e., among countries and within country. A total of 91% of the genetic variation was present within country and low variation (9%) was observed among the countries. Findings of AMOVA were also supported by the results of 100-seed weight and genetic distance. A good range of variations in 100-seed weight and genetic distance calculated at countries basis confirmed that mostly variations resulted in this study are because of diverse individuals within countries. Safflower accessions Egypt-5, Egypt-2, and India-2 showed the highest genetic distance among the studied panel and hence might be recommended as candidate parental lines for safflower breeding programs. Moreover, Egypt-5 is the only accession among the studied international safflower panel that reflected 100-seed weight of > 5.00 g at both locations and the highest genetic distance (0.76 with silicoDArT markers). Model-based structure analysis, Neighbor joining analysis and Principal coordinate analysis (PCoA) clustered the safflower accessions on the basis of their geographical origin. This is a pioneer study uncovering the marker-trait association for 100-seed weight in safflower. A total of two silicoDArT markers (DArT-45483051 and DArT-15672391) showed statistically significant association for 100-seed weight and these markers can be used in marker-assisted breeding to develop safflower cultivars with improved yield.

## Figures and Tables

**Table 1:** Analysis of variance for 100-seed weight of safflower germplasm.

**Pakistan**					
Source	**DF**	**SS**	**Mean Square**	**F Value**	**Pr > F**
Block	5	0.66	0.13	3.25	0.1108
Check	1	4.58	4.58	112.09	0.0001 **
Accessions	86	42.38	0.49	12.04	0.0053 **
Error	5	0.20	0.04		
**Turkey**					
**Source**	**DF**	**SS**	**Mean Square**	**F Value**	**Pr > F**
Block	5	0.82	0.16	6.99	0.0261
Check	1	2.13	2.13	90.86	0.0002 **
Accessions	86	30.14	0.35	14.93	0.0032 **
Error	5	0.11	0.02		

** Significant at *p* < 0.01.

**Table 2:** Minimum, maximum, mean, and standard deviation (StD) of the 100-seed weight in international safflower panel.

100-Seed Weight (g)	Minimum	Maximum	Mean	Std. Deviation
Pakistan	1.88	5.29	3.26	0.74
Turkey	2.16	5.32	3.33	0.59
Overall	2.17	5.31	3.29	0.59

**Table 3:** Diversity indices calculated to investigate genetic diversity for whole safflower panel and accessions grouped according to country of origin panel with silicoDArT markers.

Population/Country	Polymorphic Loci (%)	Na	Ne	I	He	uHe	Mean GD	GD Range
Overall population	-	1.99	1.54	0.48	0.32	0.32	0.50	0.14–0.76
Afghanistan	74.97	1.53	1.45	0.41	0.28	0.34	0.46	-
Austria	49.96	1.25	1.35	0.30	0.21	0.28	0.48	-
Bangladesh	87.37	1.74	1.57	0.48	0.33	0.37	0.44	-
China	98.44	1.98	1.66	0.56	0.38	0.41	0.46	-
Egypt	96.73	1.94	1.63	0.54	0.36	0.40	0.41	-
India	96.73	1.95	1.65	0.55	0.37	0.41	0.48	-
Iran	98.44	1.96	1.65	0.55	0.37	0.40	0.45	-
Iraq	49.90	1.24	1.35	0.30	0.21	0.28	0.42	-
Israel	87.37	1.73	1.57	0.48	0.33	0.38	0.44	-
Jordan	93.53	1.90	1.63	0.53	0.36	0.40	0.27	-
Morocco	49.90	1.23	1.34	0.30	0.20	0.26	0.42	-
Pakistan	99.81	1.98	1.69	0.58	0.39	0.42	0.44	-
Portugal	96.73	1.93	1.63	0.51	0.36	0.40	0.42	-
Spain	87.37	1.81	1.59	0.49	0.34	0.39	0.38	-
Syria	74.97	1.61	1.50	0.42	0.28	0.34	0.38	-
Turkey	99.82	1.99	1.68	0.58	0.39	0.42	0.53	-
Uzbekistan	74.97	1.62	1.52	0.43	0.29	0.35	0.48	-

Na: Observed number of alleles, Ne: Number of effective alleles, I: Shannon’s information index, He: Expected heterozygosity, uHe: Unbiased expected heterozygosity, GD: Jaccard Genetic distance.

**Table 4:** Analysis of molecular variance among countries and within country groups of safflower germplasm.

Source of Variation	Df	SS	MS	Est. Var.	% Variations
Among Countries	25	56719.33	3336.43	225.00	9
Within Country	68	165646.96	2179.56	2179.56	91
Total	93	222366.29	-	2404.57	100

**Table 5:** Marker-trait associations of the 100-seed weight with its associated markers in international safflower panel of 94 accessions.

Trait	Markers	*p*-Value	R^2^	Putative Gene
100-seed weight	DArT-45483051	1.17E-04	17.40%	*AT1G01040*
	DArT-15672391	1.15E-04	18.60%	*AT5G58040*

## Supplementary Materials

The following are available online at https://www.mdpi.com/2223-7747/9/5/652/s1, Supplementary Table S1: Mean data for 100-seed weight across two locations (Pakistan and Turkey), Supplementary Table S2: Calculated diversity parameters for the whole safflower germplasm, Supplementary Table S3: Genetic distance between the 94 safflower accessions, Supplementary Table S4: List of 94 safflower accessions evaluated to explore genetic diversity and population structure with silicoDArT molecular markers.
