# Supplementary material for: Genetic Diversity, Population Structure and Marker-Trait Association for 100-Seed Weight in International Safflower Panel Using SilicoDArT Marker Information
Source: Plants (Basel). 2020 May 21;9(5):652. doi: 10.3390/plants9050652 (PMC7284372; doi:10.3390/plants9050652)
Supplement: Supplementary file 1 [file plants-09-00652-s001.zip › plants-783153-supplementary-for proofreading/SupplementaryTable S4.docx]

**Table S4.** List of 94 safflower accessions evaluated to explore genetic diversity and population structure with silicoDArT molecular markers.

| **S.No** | **Accession Name** | **Accession No** | **Donor Organization** | **Country Origin** | **Plant ID** | **Continent** |
| --- | --- | --- | --- | --- | --- | --- |
| 1 | Afghanistan-1 | 30614 | USDA | Afghanistan | P1-253764 | Asia |
| 2 | Afghanistan-2 | 30653 | USDA | Afghanistan | P1-304592 | Asia |
| 3 | Afghanistan-3 | 33541 | USDA | Afghanistan | PI 220647 | Asia |
| 4 | Argentina-1 | 30695 | USDA | Argentina | P1-367833 | America |
| 5 | Australia-1 | 33542 | USDA | Australia | PI 235660 | Oceania |
| 6 | Austria-1 | 33568 | USDA | Austria | PI 253519 | Europe |
| 7 | Austria-2 | 33670 | USDA | Austria | BVAL-901352 | Europe |
| 8 | Bangladesh-1 | 31509 | USDA | Bangladesh | PI-401472 | Asia |
| 9 | Bangladesh-2 | 31510 | USDA | Bangladesh | PI-401478 | Asia |
| 10 | Bangladesh-3 | 31511 | USDA | Bangladesh | PI-401480 | Asia |
| 11 | Bangladesh-4 | 33609 | USDA | Bangladesh | PI 401470 | Asia |
| 12 | China-1 | 30624 | USDA | China | P1-262452 | Asia |
| 13 | China-2 | 30625 | USDA | China | P1-262453 | Asia |
| 14 | China-3 | 33638 | USDA | China | PI 543979 | Asia |
| 15 | China-4 | 33639 | USDA | China | PI 543982 | Asia |
| 16 | China-5 | 33642 | USDA | China | PI 544001 | Asia |
| 17 | China-6 | 33651 | USDA | China | PI 568809 | Asia |
| 18 | China-7 | 33661 | USDA | China | PI 568874 | Asia |
| 19 | Egypt-1 | 30563 | USDA | Egypt | P1-250082 | Africa |
| 20 | Egypt-2 | 30574 | USDA | Egypt | P1-250528 | Africa |
| 21 | Egypt-3 | 30577 | USDA | Egypt | P1-250532 | Africa |
| 22 | Egypt-4 | 30578 | USDA | Egypt | P1-250540 | Africa |
| 23 | Egypt-5 | 30580 | USDA | Egypt | P1-250605 | Africa |
| 24 | Egypt-6 | 30581 | USDA | Egypt | P1-250608 | Africa |
| 25 | France-1 | 33662 | USDA | France | PI 576985 | Europe |
| 26 | Hungary-1 | 33575 | USDA | Hungary | PI 288983 | Europe |
| 27 | India-1 | 30579 | USDA | India | P1-250601 | Asia |
| 28 | India-2 | 30662 | USDA | India | P1-305195 | Asia |
| 29 | India-3 | 30673 | USDA | India | P1-306926 | Asia |
| 30 | India-4 | 30674 | USDA | India | P1-306941 | Asia |
| 31 | India-5 | 30677 | USDA | India | P1-306976 | Asia |
| 32 | India-6 | 33538 | USDA | India | PI 199878 | Asia |
| 33 | Iran-1 | 30588 | USDA | Iran | P1-250720 | Asia |
| 34 | Iran-2 | 30631 | USDA | Iran | P1-304444 | Asia |
| 35 | Iran-3 | 30633 | USDA | Iran | P1-304448 | Asia |
| 36 | Iran-4 | 30713 | USDA | Iran | P1-405958 | Asia |
| 37 | Iran-5 | 30718 | USDA | Iran | P1-405967 | Asia |
| 38 | Iran-6 | 33556 | USDA | Iran | PI 250840 | Asia |
| 39 | Iran-7 | 33621 | USDA | Iran | PI 406010 | Asia |
| 40 | Israel-1 | 30548 | USDA | Israel | P1-198990 | Asia |
| 41 | Israel-2 | 30594 | USDA | Israel | P1-253386 | Asia |
| 42 | Israel-3 | 3015 | USDA | Israel | P1-253892 | Asia |
| 43 | Israel-4 | 33564 | USDA | Israel | PI 251290 | Asia |
| 44 | Iraq-1 | 30612 | USDA | Iraq | P1-253761 | Asia |
| 45 | Iraq-2 | 30613 | USDA | Iraq | P1-253762 | Asia |
| 46 | Jordan-1 | 30589 | USDA | Jordan | P1-251284 | Asia |
| 47 | Jordan-2 | 30590 | USDA | Jordan | P1-251285 | Asia |
| 48 | Jordan-3 | 33559 | USDA | Jordan | PI 251265 | Asia |
| 49 | Jordan-4 | 33560 | USDA | Jordan | PI 251267 | Asia |
| 50 | Jordan-5 | 33561 | USDA | Jordan | PI 251268 | Asia |
| 51 | Kazakhstan-1 | 30681 | USDA | Kazakhstan | P1-314650 | Asia |
| 52 | Libya-1 | 33608 | USDA | Libya | PI 393499 | Africa |
| 53 | Morocco-1 | 30552 | USDA | Morocco | P1-239042 | Africa |
| 54 | Morocco-2 | 30606 | USDA | Morocco | P1-253560 | Africa |
| 55 | Pakistan-1 | 30564 | USDA | Pakistan | P1-250194 | Asia |
| 56 | Pakistan-2 | 30565 | USDA | Pakistan | P1-250201 | Asia |
| 57 | Pakistan-3 | 30567 | USDA | Pakistan | P1-250345 | Asia |
| 58 | Pakistan-4 | 30568 | USDA | Pakistan | P1-250346 | Asia |
| 59 | Pakistan-5 | 30569 | USDA | Pakistan | P1-250351 | Asia |
| 60 | Pakistan-6 | 30570 | USDA | Pakistan | P1-250353 | Asia |
| 61 | Pakistan-7 | 30573 | USDA | Pakistan | P1-250481 | Asia |
| 62 | Pakistan-8 | 33547 | USDA | Pakistan | PI 250474 | Asia |
| 63 | Pakistan-9 | 33548 | USDA | Pakistan | PI 250478 | Asia |
| 64 | Pakistan-10 | 33635 | USDA | Pakistan | PI 426521 | Asia |
| 65 | Pakistan-11 | Check | PGRI-Pakistan | Pakistan | Thori-78 | Asia |
| 66 | Portugal-1 | 30604 | USDA | Portugal | P1-253553 | Europe |
| 67 | Portugal-2 | 30605 | USDA | Portugal | P1-253556 | Europe |
| 68 | Portugal-3 | 30608 | USDA | Portugal | P1-253564 | Europe |
| 69 | Portugal-4 | 30610 | USDA | Portugal | P1-253569 | Europe |
| 70 | Portugal-5 | 30611 | USDA | Portugal | P1-253571 | Europe |
| 71 | Portugal-6 | 30620 | USDA | Portugal | P1-258412 | Europe |
| 72 | Romania-1 | 30549 | USDA | Romania | P1-209287 | Europe |
| 73 | Russia-1 | 30663 | USDA | Russia | P1-305535 | Asia |
| 74 | Spain-1 | 30595 | USDA | Spain | P1-253388 | Europe |
| 75 | Spain-2 | 30596 | USDA | Spain | P1-253391 | Europe |
| 76 | Spain-3 | 30597 | USDA | Spain | P1-253394 | Europe |
| 77 | Spain-4 | 30598 | USDA | Spain | P1-253395 | Europe |
| 78 | Syria-1 | 30616 | USDA | Syria | P1-253898 | Asia |
| 79 | Syria-2 | 30617 | USDA | Syria | P1-253900 | Asia |
| 80 | Syria-3 | 30700 | USDA | Syria | P1-386174 | Asia |
| 81 | Thailand-1 | 30701 | USDA | Thailand | P1-387821 | Asia |
| 82 | Turkey-1 | 30646 | USDA | Turkey | P1-304498 | Asia |
| 83 | Turkey-2 | 30648 | USDA | Turkey | P1-304502 | Asia |
| 84 | Turkey-3 | 30650 | USDA | Turkey | P1-304504 | Asia |
| 85 | Turkey-4 | 30651 | USDA | Turkey | P1-304505 | Asia |
| 86 | Turkey-5 | 30688 | USDA | Turkey | P1-340086 | Asia |
| 87 | Turkey-6 | 33543 | USDA | Turkey | PI 237538 | Asia |
| 88 | Turkey-7 | 33565 | USDA | Turkey | PI 251978 | Asia |
| 89 | Turkey-8 | 33567 | USDA | Turkey | PI 251984 | Asia |
| 90 | Turkey-9 | 33627 | USDA | Turkey | PI 406701 | Asia |
| 91 | Turkey-10 | Check | USDA | Turkey | Dinçer | Asia |
| 92 | Uzbekistan-1 | 30623 | USDA | Uzbekistan | P1-262435 | Asia |
| 93 | Uzbekistan-2 | 30696 | USDA | Uzbekistan | P1-369846 | Asia |
| 94 | Uzbekistan-3 | 30697 | USDA | Uzbekistan | P1-369853 | Asia |

USDA: United States Department of Agriculture; PGRI: Plant Genetic Resources Institute; Pakistan-11 and Turkey-10: Commercial cultivars.
